# Small-Pox Cases at a Public Meeting

**Published:** 1888-03-03

**Authors:** Wm. J. Clegg

**Affiliations:** Mayor


### The Editor's Letter-Box.

[Our Correspondents are reminded that prolixity is a great bar to publication, and that brevity of style and conciseness of statement greatly facilitate early publication.]

### SMALL-POX CASES AT A PUBLIC MEETING.

My attention has been called to a paragraph in your paper of the 25th ult., in which you state that, in reference to the meeting as to small-pox held at Sheffield, over which I presided, that "we are told that many of those present were suffering from small-pox." So far as I know, and as I said before I presided at the meeting, there was no one suffering from small-pox present at such meeting, neither have I heard of any such being present. It appears to me astonishing that conductors of newspapers should go out of their way, as they appear to do, to malign the town of Sheffield because it happens to be suffering from the small-pox epidemic.

Wm. J. Clegg, Mayor.

[Our report of the meeting referred to was taken from a local paper, sent to us from Sheffield.—Ed.]

